# First prospective data on breast cancer patients from the multicentre italian bone metastasis database

**DOI:** 10.1038/s41598-021-83749-1

**Published:** 2021-02-22

**Authors:** Alberto Bongiovanni, Flavia Foca, Manuela Fantini, Maria Rosachiara Forcignanò, Fabrizio Artioli, Rossana Berardi, Enrico Campadelli, Giuseppe Procopio, Francesco Silvestris, Nada Riva, Lorena Gurrieri, Silvia Angela Debonis, Giandomenico Di Menna, Valentina Fausti, Federica Recine, Roberto Vespignani, Toni Ibrahim

**Affiliations:** 1 Osteoncology and Rare Tumors Center (CDO-TR), IRCCS Istituto Romagnolo per lo Studio dei Tumori (IRST) “Dino Amadori”, Meldola, Italy; 2 Unit of Biostatistics and Clinical Trials, IRCCS Istituto Romagnolo per lo Studio dei Tumori (IRST) “Dino Amadori”, Via P. Maroncelli 40, 47014 Meldola, Italy; 3 Oncology Unit, Infermi Hospital, Rimini, Italy; 4 Oncological Unit, Vito Fazzi Hospital, Lecce, Italy; 5 Division of Medical Oncology, Ramazzini Hospital, Carpi, Italy; 6 Ospedali Riuniti di Ancona, Ancona, Italy; 7 Oncology Unit, Degli Infermi Hospital, Faenza, Italy; 8 IRCCS National Cancer Institute (INT), Milan, Italy; 9 Department of Biomedical Science and Human Oncology, University of Bari, Bari, Italy; 10 IT Service, IRCCS Istituto Romagnolo per lo Studio dei Tumori (IRST) “Dino Amadori”, Meldola, Italy

**Keywords:** Cancer, Breast cancer, Metastasis

## Abstract

Bone metastases (BM) are still the main cause of morbidity in cancer patients because of skeletal-related events (SREs) that reduce quality of life. They have also led to increased social and healthcare costs. At present, data available on BM are insufficient. This was a multicentre prospective observational study of patients with BM from breast cancer (BC) with at least 6 months’ follow-up. Information on patients at the first diagnosis of BM, including demographics and characteristics of the primary tumor and BM. Data were periodically updated by participating centres and reviewed by the coordinator centre. From October 2014 to July 2019, 618 patients with BM from solid tumors were enrolled and 220 were eligible for the present study. Median age was 62 years (range 26–86). Median follow-up was 34 months (range 6–149). At the time of enrolment, 109 (50%) had only BM (BOM) and 109 (50%) had concomitant visceral lesions and BM (BVM). Median time-to-first BM was 47 months (range 0–312) in BOM and 78.6 months in BVM patients. Disease-free interval differed on the basis of BC molecular subtype and stage. Ninety-eight BM patients had at least on SRE. Zoledronate was used in 69.1% of cases and denosumab in 28.3%. First-line treatment was hormone-based (50.7%), chemotherapy-based (38.7%) or chemotherapy- + hormone therapy-based (9.7%). Median progression-free and overall survival were 15.1 months (95% CI 12.6–18.4) and 66.8 months (95% CI 52.1–79.2), respectively. Our prospective study could substantially help to better understand the natural history of BM from BC.

## Introduction

Breast cancer (BC) is the most common malignancy and a major cause of morbidity and mortality among women. The mortality rate has decreased thanks to improved diagnostic procedures, screening and more advanced treatments. However, the rate of recurrence in distant organs is still fairly high, ranging from 20 to 30%^1,2^.

Bone is the most common site of metastasis in BC and significantly impacts patient survival^3–5^.

Bone metastases (BMs) represent an important clinical-epidemiological issue in oncology because their diagnosis and treatment are often necessarily handled by several specialists, resulting in fragmented patient information^6^. For these reasons, great efforts have been made to develop a new scientific and clinical branch of medicine, i.e. Osteoncology^7^.

The major problem faced by BM patients is the risk of skeletal complications defined as skeletal-related events (SREs) all of which are highly detrimental to quality of life and survival^2,8,9^.

There is still limited information available on BM clinical presentation, the difference in disease response between bone and visceral sites, and the difference in prognosis between solitary, oligometastatic and multiple sites or axial and trunk bone metastases^10^. A clearer understanding of their natural evolution would thus help us to identify new strategies capable of reducing both BM incidence and morbidity.

The risk of SREs in BC patients with BM has been the focus of numerous studies^11–13^. However, their findings are of limited value because of their poor generalizability with respect to current clinical practice. In the retrospective studies, authors usually considered a lengthy time period during which available therapies and clinical practice may have changed substantially. In the prospective studies, patients were followed for a short period (24 months) and data were extrapolated from a BC database rather than from a database dedicated to BM. Furthermore, in recent years, new therapeutic options have become available. There has also been growing interest in BM since dedicated multidisciplinary groups began to emerge^14^.

The main aims of this prospective multicenter study were to evaluate the evolution of skeletal disease in BC patients, assess the impact of BM on disease outcome, examine the role of a number of clinical-pathological parameters in predicting survival, and further our understanding of the natural history of patients with BM from BC.

## Materials and methods

This was a multicentre prospective observational study of patients with BM from BC with at least 6 months follow-up, enrolled into the prospective Italian Bone Metastases Data Base (BMDB). The study was approved by the Local Ethics Committee of each participating centre and carried out in accordance with the ethical standards laid down in the 1964 Declaration of Helsinki. List of participating centers was provided in Supplementary Table 1. Written informed consent was obtained from all patients. Information on data source is provided in Appendix 1.

### Data extraction and measure definition

The evolution of skeletal disease in BC patients was evaluated by extracting data from the BMDB for patients who had a first diagnosis of BC with a synchronous (within 2 months) or metachronous diagnosis of bone metastasis and were followed up for at least 6 months after the BM diagnosis.

Time to event outcomes were defined as follows: disease-free interval (DFI) was the time from primary BC disease to the appearance of the first metastasis (bone or visceral), and bone disease-free interval (bDFI) was the time between diagnosis of primary BC and first diagnosis of BM. Overall survival (OS) was calculated as the time from the date of the diagnosis of primary BC to the date of death. OS from metastatic disease (metOS) was calculated as the time from the diagnosis of metastasis (either bone or visceral) to death. Progression-free survival (PFS) was the time between the date of the first diagnosis of bone metastasis and date of the first documented evidence of disease progression (bone or visceral) and death. Bone PFS (bPFS) was the time between the date of the first diagnosis of BM and first progression to bone and death. Time-to-first SRE was the time between the first diagnosis of BM and the first SRE event. Patients without events of interest were censored at the date of the last follow-up visit.

### Statistical analysis

Descriptive statistics are used to summarize baseline patient characteristics, BM characteristics and treatment patterns. Continuous variables are presented using median and range or interquartile range. The Wilcoxon rank-sum test was used for continuous variables, together with the chi-squared test or Fisher’s exact test, as appropriate. McNemar’s test was used in cases of paired data. Time-to-event measures were analysed using the Kaplan–Meier method, and the nonparametric log-rank test was used to evaluate the role of stratification factor. We used the Cox proportional hazards regression model to estimate hazard ratios (HRs) and relative 95% confidence intervals (CI) of potential clinical prognostic factors for time-to-event outcomes.

All statistical analyses were performed using STATA/MP 15.0 for Windows (StataCorp LLP, College Station, TX, USA).

## Results

### Patient characteristics

**Figure 1:**
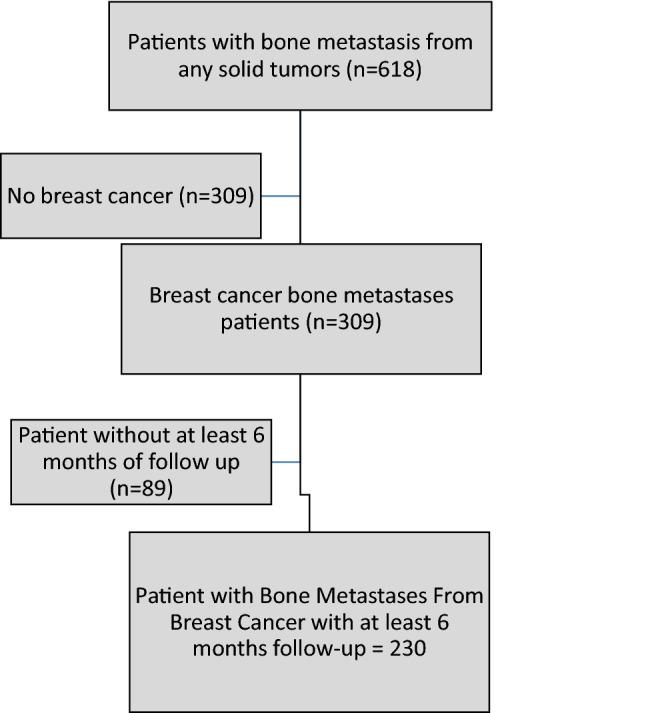
Study flow diagram.

From October 1st 2014 to June 30th 2018, 618 patients with BM from any solid tumor were registered in the Italian BMDB. Three hundred and nine had BC as the primary site of disease and 220/309 with at least 6 months’ follow up were included in the present analysis (Fig. 1). Median age was 62 (range 26–85) years.

At the time of the first diagnosis of BM, 152 (92.1%) patients showed a good ECOG PS (0–1). Forty-nine (22.3%) patients were diagnosed with BM synchronous to the primary tumor, while metachronous BM were found in 171 patients.

Bone-only metastases (BOM) were found in 109 (50.0%) patients, while the remaining 109 had concomitant visceral and BM (BVM). Histological and biological characteristics of the primary BC are shown in Tables 1 and 2. Luminal A and B tumors were more frequently associated with BOM, whereas basal-like or HER2-enriched BC subtypes more often showed BVM (p = 0.012). A higher, albeit not significant, Ki-67 value was observed for patients with BVM (p = 0.074). The majority of patients had T0-T2 (*n* = 159, 85%) and node-positive tumors (*n* = 138, 74.6%) at diagnosis (Table 3), the former associated with a higher rate of metachronous BM than synchronous BM (83.2% and 16.8%, respectively) (p < 0.001) (Table 4). Patients with N0 primary tumors had a higher incidence of metachronous BM than synchronous (95.8% and 4.2%, respectively) (p = 0.001). No difference between BOM or BVM according to node status (node negative *vs*. node positive tumors) was observed. Both node-negative and node-positive patients showed a high rate of metachronous BM (95.8% and 75.5%, respectively), even if in node-negative patients there is a significantly higher proportion of patients with metachronous BM.

**Table 1:** Patient characteristics at baseline and at onset of BM.

	Patients (n = 220)
**Median age, years (range)**	62 (26–85)
	**No. (%)**
**Age at diagnosis of primary BM, years**	
< 65	133 (60.5)
65	87 (69.5)
**ECOG PS at diagnosis of primary BM**	
0–1	152 (92.1)
≥ 2	13 (7.9)
Unknown	55
**Histology**	
Ductal carcinoma	166 (75.5)
Lobular carcinoma	29 (13.0)
Mixed ductal and lobular carcinoma	11 (5.0)
Adenocarcinoma, NOS	9 (4.0)
Signet ring cell carcinoma	1 (0.5)
Other	4 (2.0)
**pT at primary diagnosis of BC**	
T0–T2	159 (85.0)
T3–T4	28 (15.0)
Tx	31
**pN at primary diagnosis of BC**	
N0	47 (25.4)
N+	138 (74.6)
Nx	33
**Stage at diagnosis of primary disease**	
I	28 (14.1)
II	68 (34.3)
III	42 (21.2)
IV	60 (30.3)
Unknown	22
**BC molecular subtype**	
Luminal A	35 (18.8)
Luminal B	118 (63.4)
Basal-like	8 (4.3)
HER+	25 (13.4)
Unknown	34
**Grading**	
G1	6 (3.7)
G2	85 (52.5)
G3	71 (43.8)
Unknown	58
**Bone metastasis**	
Synchronous	49 (22.3)
Metachronous	171 (77.7)
BOM	109 (50.0)
BVM	109 (50.0)

*BM* bone metastasis, *ECOG PS* Eastern Cooperative Oncology Group Performance Status, *NOS* not otherwise specified, *BC* breast cancer, *pT* primary tumour, *pN* pathological lymph node, *Nx* unknown lymph node stage, *G* grade, *BOM* bone-only metastasis, *BVM* visceral and bone metastasis.

**Table 2:** Biomarker characteristics at diagnosis of primary BC and at onset of BM.

BC characteristics	At diagnosis			At the onset of bone metastasis		
	Positive* (%)	Negative* (%)	**NA**	Positive* (%)	Negative* (%)	**NA**
ER	183 (89.7)	21 (10.3)	16	48 (88.9)	6 (11.1)	166
PgR	144 (70.6)	60 (29.4)	16	24 (45.3)	29 (54.7)	167
	**< 15%**	**≥ 15%**	**NA**	**< 15%**	**≥ 15%**	**NA**
Ki-67	145 (79.7)	37 (20.3)	38	29 (65.9)	15 (34.1)	176
	**Positive/(+ + +)**	**Negative/0–2 + **	**NA**	**Positive/(+ + +)**	**Negative/0–2 + **	**NA**
HER2 (IHC or FISH)	24 (12.9)	162 (87.1)	34	7 (13.7)	44 (86.3)	169

*BC* breast cancer, *BM* bone metastasis, *ER* oestrogen receptor, *PgR* progesterone receptor, *NA* not available, *IHC* immunohistochemistry, *FISH* fluorescence in situ hybridisation.

*For ER and PgR, positive if ≥ 10%, negative if < 10%.

**Table 3:** Baseline patient characteristics in relation to presence of visceral metastases.

Baseline BC characteristics	No. patients (n = 218)	BOM (n = 109) No. (%)	BVM (n = 109) No. (%)	p-value
**Age at diagnosis of bone metastasis, years**				
< 65	108 (49.1)	70 (53.0)	62 (47.0)	0.268
≥ 65	112 (50.9)	39 (45.3)	47 (54.7)	
**pT at primary diagnosis of breast cancer**				
T0–T2	159 (85.0)	84 (52.8)	75 (47.2)	0.531
T3–T4	28 (15.0)	13 (46.4)	15 (53.6)	
**pN at primary diagnosis of breast cancer**				
N0	47 (25.4)	26 (55.3)	21 (44.7)	0.330
N+	138 (74.6)	65 (47.1)	73 (52.9)	
**Stage at diagnosis of primary disease**				
I	27 (13.8)	16 (59.3)	11 (40.7)	0.621
II	67 (34.2)	31 (46.3)	36 (53.7)	
III	42 (21.4)	23 (54.8)	19 (45.2)	
IV	60 (30.6)	33 (55.0)	27 (45.0)	
Unknown	22	6	16	
**Breast cancer molecular subtype**				
Luminal A	35 (19.0)	26 (74.3)	9 (25.7)	0.012
Luminal B	118 (64.1)	60 (50.8)	58 (49.2)	
Basal-like	7 (3.8)	2 (28.6)	5 (71.4)	
HER+	24 (13.1)	9 (37.5)	15 (62.5)	
Unknown	34	12	22	
**Median Ki67% (interquartile range)**	20 (10–31)	16 (8–30)	20 (10–35)	0.074
**ER**				
Negative	19 (9.4)	6 (31.6)	13 (68.4)	0.075
Positive	183 (90.6)	97 (53.1)	86 (46.9)	
Unknown or not performed	16	6	10	
**PgR**				
Negative	58 (28.7)	29 (50.0)	29 (50.0)	0.858
Positive	144 (71.3)	74 (51.4)	70 (48.6)	
Unknown or not performed	16	6	10	
**HER2 (IHC or FISH)**				
Negative	161 (87.5)	89 (55.3)	72 (44.7)	0.066
Positive	23 (12.5)	8 (34.8)	15 (65.2)	
Unknown or not performed	34	12	22	
**Adjuvant therapy**				
No	73 (33.4)	35 (47.9)	38 (52.1)	0.632
Yes	144 (66.1)	74 (51.4)	70 (48.6)	
**Neoadjuvant therapy**				
No	196 (91.2)	97 (49.5)	99 (50.5)	0.484
Yes	19 (8.8)	11 (57.9)	8 (42.1)	

*BC* breast cancer, *BOM* bone-only metastasis, *BVM* visceral and bone metastasis, *pT* primary tumour, *pN* pathological lymph node, *ER* oestrogen receptor, *PgR* progesterone receptor, *IHC* immunohistochemistry, *FISH* fluorescence in situ hybridisation.

**Table 4:** Baseline patient characteristics in relation to synchronous or metachronous metastases.

Baseline BC characteristics	No. patients (n = 220)	Synchronous BM (n = 49) No. (%)	Metachronous BM (n = 171) No. (%)	p-value
**Age at diagnosis of bone metastasis, years**				
< 65	133 (49.1)	32 (24.1)	101 (75.9)	0.431
≥ 65	87 (50.9)	17 (19.5)	70 (80.5)	
**pT at primary diagnosis of breast cancer**				
T0–T2	161 (85.0)	27 (16.8)	134 (83.2)	< 0.001
T3–T4	28 (15.0)	15 (53.6)	13 (46.4)	
**pN at primary diagnosis of breast cancer**				
N0	48 (25.6)	2 (4.2)	46 (95.8)	0.001
N+	137 (74.3)	34 (24.5)	105 (75.5)	
**Stage at diagnosis of primary disease**				
I	28 (14.1)	0 (0.0)	28 (100.0)	–
II	68 (34.4)	0 (0.0)	68 (100.0)	
III	42 (21.2)	0 (0.0)	42 (100.0)	
IV	60 (30.3)	49 (81.7)	11 (18.3)	
Unknown	22	–	22	
**Breast cancer molecular subtype**				
Luminal A	35 (18.8)	7 (20.0)	28 (80.0)	0.315
Luminal B	118 (63.4)	31 (26.3)	87 (73.7)	
Basal-like	8 (4.3)	1 (12.5)	7 (87.5)	
HER+	25 (13.4)	10 (40.0)	15 (60.0)	
Unknown	34	–	34	
**Median Ki67% (interquartile range)**	20 (10–31)	23 (15–35)	16 (10–30)	0.087
**ER**				
Negative	21 (9.9)	2 (9.5)	19 (90.5)	0.114
Positive	183 (90.1)	47 (25.7)	136 (74.3)	
Unknown or not performed	16	–	16	
**PgR**				
Negative	60 (29.4)	11 (18.3)	49 (81.7)	0.220
Positive	144 (70.6)	38 (26.4)	106 (73.6)	
Unknown or not performed	16	–	16	
**HER2 (IHC or FISH)**				
Negative	162 (87.1)	38 (23.5)	124 (76.5)	0.057
Positive	24 (12.9)	10 (41.7)	14 (58.3)	
Unknown or not performed	34	1	33	

*BM* bone metastasis, *pT* primary tumour, *pN* pathological lymph node, *ER* oestrogen receptor, *PgR* progesterone receptor, *IHC* immunohistochemistry, *FISH* fluorescence in situ hybridisation.

Bone biopsy was performed in 58 (26.4%) cases. The median time from primary disease diagnosis to the appearance of BM in this subgroup was 79 months (95%CI: 65.0–118.1).

#### Time to event outcomes

##### Disease-free interval

Disease free-interval was calculated excluding patients with synchronous disease at bone (n = 49) and visceral (n = 2). The disease-free interval (DFI) differed slightly according to molecular subtype. The univariate hazard ratio (HR) for visceral or bone metastasis was higher in luminal B tumors (1.66, 95% confidence interval [CI] 1.1–2.5) (p = 0.023), basal-like tumors (3.92, 95% CI 1.6–9.7) (p = 0.003), and HER2-enriched tumors (1.28, 95% CI 0.7–2.4) (p = 0.442).

DFI for patients with stage I disease at diagnosis of primary BC was longer than that for stage III patients (median 67.2 months, 95% CI 53.1–96.1, *vs*. 58.1 months, 95% CI 41.9–73.4), with a univariate HR of 1.84 (95% CI 1.1–3.0) (p = 0.015) for the stage III group, and 0.98 (95% CI 0.6–1.5) (p = 0.931) for the stage II group. Older patients had a higher risk of metastasis (HR 1.91, 95% CI 1.4–2.7), as did those with larger tumors at diagnosis (HR: 3.7, 95% CI 1.9–7.1). Multivariate analysis confirmed these data for patients with basal-like and larger tumors (Table 5).

**Table 5:** Median DFI and independent risk factors for metastasis.

	Overall DFI			bDFI		
	Median (95%CI)	HR from univariate Cox regression model (95%CI)	HR from multivariate Cox regression model (95%CI)	Median (95%CI)	HR from univariate Cox regression model (95%CI)	HR from multivariate Cox regression model (95%CI)
**All cases**	75.7 (63.5–87.3)	–	–	78.2 (63.6–87.9)	–	–
**Age at diagnosis of primary BC, years**						
< 55	82.1 (65.4–112.9)	1.00	1.00	89.6 (65.4–114.8)	1.00	1.00
≥ 55	65.0 (48.1–81.5)	1.91 (1.4–2.7)	1.48 (0.9–2.2)	65.0 (51.1–86.0)	1.89 (1.4–2.6)	1.27 (0.9–1.9)
**BC molecular subtypes**						
Luminal A	101.9 (57.0–125.7)	1.00	1.00	101.9 (57.0–125.7)	1.00	1.00
Luminal B	63.5 (48.6–75.7)	1.66 (1.1–2.5)	1.46 (0.9–2.4)	63.6 (52.7–78.5)	1.54 (0.9–2.4)	1.27 (0.8–2.0)
Basal-like	30.0 (13.4–NE)	3.92 (1.6–9.7)	3.94 (1.4–11.0)	30.0 (2.1–66.1)	4.29 (1.8–10.1)	3.82 (1.4–10.6)
HER2+	53.1 (30.9–100.1)	1.28 (0.7–2.4)	1.44 (0.7–2.9)	53.1 (47.9–100.2)	1.17 (0.6–2.2)	1.22 (0.6–2.5)
**Stage at diagnosis**						
I	67.2 (53.1–96.1)	1.00	1.00	82.2 (53.6–125.7)	1.00	1.00
II	83.3 (66.5–99.2)	0.98 (0.6–1.5)	1.09 (0.5–2.3)	83.2 (66.5–101.9)	1.07 (0.7–1.7)	1.16 (0.6–2.4)
III	58.1 (41.9–73.4)	1.84 (1.1–3.0)	1.35 (0.5–3.2)	60.9 (41.9–75.6)	2.1 (1.3–3.5)	1.55 (0.7–3.7)
IV	–	–	–	–	–	–
**pT at primary diagnosis of BC**						
T0–T2	76.7 (61.0–87.3)	1.00	1.00	78.5 (63.6–87.9)	1.00	1.00
T3–T4	20.6 (3.3–65.4)	3.7 (1.9–7.1)	3.24 (1.5–7.1)	18.6 (3.3–57.0)	4.5 (2.4–8.2)	3.02 (1.4–6.6)
**pN at primary diagnosis of BC**						
N0	83.3 (56.1–101.9)	1.00	1.00	89.6 (61.1–118.2)	1.00	1.00
N+	66.5 (56.3–80.0)	1.36 (0.9–1.9)	0.91 (0.5–1.7)	66.5 (57.0–79.0)	1.47 (1.0–2.1)	0.97 (0.5–1.8)

*DFI* disease-free interval, *bDFI* bone disease-free interval, *HR* hazard ratio, *BC* breast cancer, *NE* not evaluable from statistical software, *pT* primary tumour, *pN* pathological lymph node.

##### Bone disease-free interval

For this analysis, were excluded all patients with synchronous bone metastasis (n = 49). Median time to BM appearance was 78.2 months (95% confidence interval [CI] 63.6–87.9) for all patients.

**Figure 2:**
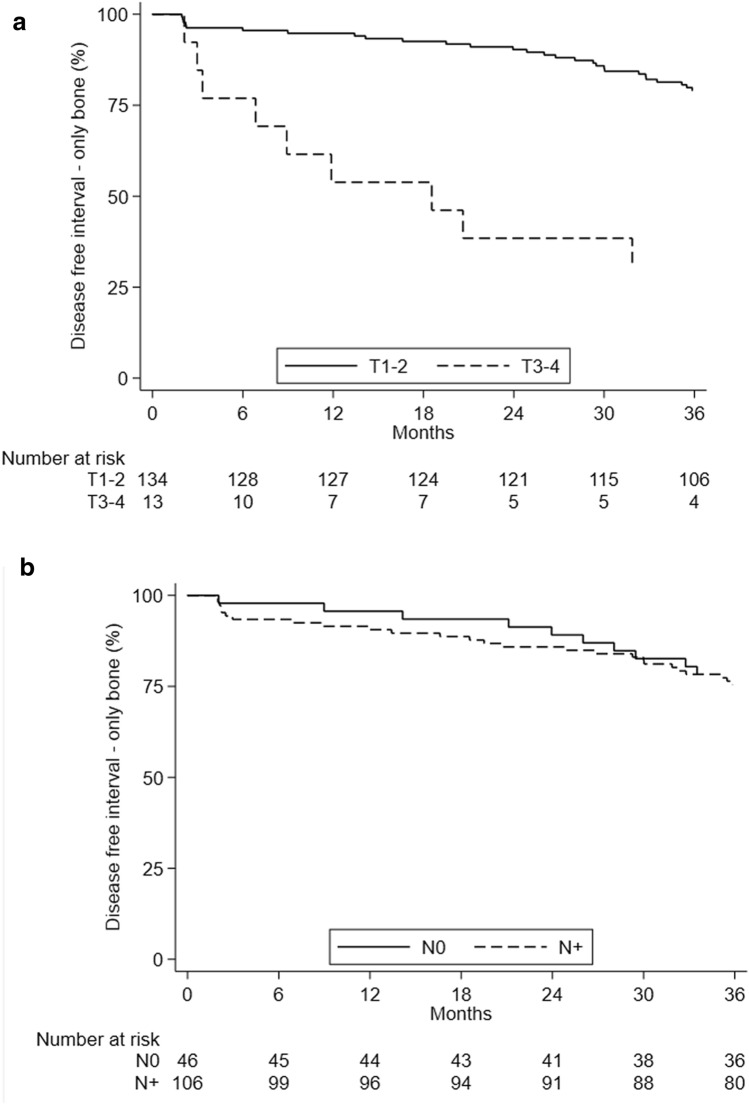
Disease-free interval by (**a**) T and (**b**) N of primary disease.

Median bone disease-free interval (bDFI) was 63.5 months (95% CI 47.9–83.3) for the BM-only group at diagnosis and 86.6 months (95% CI 66.5–99.6) for those with visceral metastases. Median bDFI was 78.5 months (95% CI 63.6–87.9) in T0–T2 patients and 18.6 months (95% CI 3.3–57.0) in the T3–T4 group (p ≤ 0.001). The group with node-negative BC had a median bDFI of 8.6 months (95% CI 61.1–118.2) compared to 66.5 months (95% CI 57.0–79.0) for the node-positive subgroup (log rank test p = 0.032) (Fig. 2a,b). bDFI was significantly higher (p < 0.001) in patients aged < 55 years at diagnosis than in those ≥ 55 years, (median 89.6 [95% CI 65.4–114.8] *vs*. 65.0 [95% CI 51.1–86.0] months) (Supplementary Fig. 1). Multivariate analyses confirmed a higher risk for patients with basal-like and larger tumors (Table 5).

#### Overall survival

Median follow-up was 46 months (range: 6–117) on 220 evaluable patients.

Seventy-four deaths were observed during follow-up. Median OS was 217.5 months (95% CI 172.5–340.1).

Molecular profile subtypes were an independent prognostic factor. Median OS (mOS) in patients with luminal A tumors was not-reached and 128.1 months (95% CI 108.0–182.6) for those with luminal B tumors, 101.2 months (95% CI 17.1–not estimable) for patients with basal-like BC, and 274.5 months (95% CI 70.3-–not estimable) for those HER2-enriched BC (p = 0.010). Patients aged ≥ 55 years and those with stage IV disease at diagnosis had a shorter mOS (128.1 months [95% CI 101.2–182.6] and 65.3 months [95% CI 41.0–80.9], respectively) than the groups diagnosed at a younger age (< 55 years) and with lower-stage disease (Supplementary Fig. 2a,b). Patients with pain at the first diagnosis of bone metastases had an mOS of 143.8 months (95% CI 98.0–247.5) with respect to 257.4 months (95% CI 135.1–not estimable) for those with no pain. mOS of the group with axial BM was 252.5 months (95% CI 182.5–343.0), 157.6 months (95% CI41.0–Not estimable) for those with appendicular BM, and 217.5 months (95% CI 100.9–Not estimable) for patients with both types of metastases (p = 0.009).

mOS of patients undergoing first-line treatment was 135.1 months (95% CI 102.9–257.4) for the chemotherapy (CH) ± biological therapy (BIO) group and 252.5 months (95%CI:202.1-not estimable) for those receiving endocrine therapy (ENDO) ± BIO, but was not-reached in patients undergoing CH + ENDO (p = 0.0305). Patients aged ≥ 55 years (HR 2.92, 95% CI 1.4–6.0), those with luminal B (HR 4.10, 95% CI 1.5–11.1), stage IV disease (HR 8.69, 95% CI 2.6–28.9) or axial + appendicular or other site of BM (HR:2.20, 95% CI 1.1–4.6) had a higher risk of death, while those with no pain at BM diagnosis (HR 0.49, 95% CI 0.2–0.9) and patients receiving ENDO ± BIO (HR:0.40, 95% CI 0.2–0.8) had a better prognosis considering multivariate Cox regression model (Supplementary Table 2).

#### OS from diagnosis of metastatic disease

The median OS for patients with metastatic disease (metOS) was 66.8 months (95% CI 52.1–79.2). The molecular profile of subtypes was an independent prognostic factors according to metOS. A multivariate Cox regression model confirmed a poorer prognosis for patients with luminal B subtype (HR 3.67, 95% CI 1.5–8.7) (Supplementary Table 2).

#### Progression-free survival

Disease progression occurred in 167 patients. Median progression-free survival (PFS) was 15.1 months (95% CI 12.6–18.4). With respect to first-line treatment, median PFS was 13.4 months (95% CI 10.0–16.6) in the CH ±BIO arm, 17.3 months (95% CI 12.0–23.6) in the ENDO ± BIO group and 32.0 months (95% CI 12.7-Not estimable) in CH + ENDO patients. None of analyzed prognostic factors were found as statistically significant in univariate analysis, even if a HR of 0.51 (95% CI 0.27–0.95) was observed for patients treated with CH + ENDO with respect to those given CH alone after the first diagnosis of metastasis (p = 0.0849) (Supplementary Fig. 3) in univariate analysis. No differences in terms of time to disease progression were seen between synchronous and metachronous BM, BOM vs. BVM, first-line treatment, number and type of BM, and presence of pain at diagnosis. The presence of SREs at diagnosis did not have an impact on disease progression (Supplementary Table 3).

#### Bone metastasis progression-free survival

Median BM PFS was 45.9 months (95% CI 30.8–63.0). Older patients had a higher risk for progression to bone (HR: 1.51, 95% CI 1.1–2.1) in univariate analysis (Supplementary Table 3).

#### Time to first SRE

Ninety-eight (44.5%) patients had at least one SRE during the course of their metastatic disease. Patients treated with zoledronic acid or pamidronate had a similar HR for SREs with respect to untreated patients (HR 1.32, 95% CI 0.74–2.38), while those taking denosumab had a HR of 0.20 (95% CI 0.04–0.87), indicating a reduced risk of SRE (Supplementary Fig. 4). Supplementary data are reported in Appendix 2.

## Discussion

Bone metastases represent a common complication of cancer, their incidence reaching around 65% in BC^2^. There are still aspects of bone metastatic disease that need to be further investigated^15,16^.

As reported in previous studies, our case series showed a majority of lytic bone metastases^17,18^. The nature (lytic or not) of BM would not appear to impact patient outcome. Small BC tumors (T0–T2) were associated with metachronous BM, with a time to bone involvement of 65.1 months after the primary diagnosis of BC, indicating that the information collected also regarded patients with latent BM. This subgroup possesses the clinical phenotype of bone metastatic cells characterized by dormancy in which adjuvant BTT could prove useful to prevent BM formation^19^. The mOS from the diagnosis of distant disease (5.5 years) was similar to that reported in other studies, whereas mOS from the primary BC diagnosis differed^20–22^. Such findings reflect the good prognosis of the primary BC patients included in our study, representing a real-world population.

Recent studies on a population-based cancer registry and a National Cancer Database observed that patients aged < 60 years now show better survival than those reported in previous studies in which younger age and premenopausal status were associated with poorer survival^22–24^. Our study had similar findings, with improved mOS from the time of diagnosis of metastastic disease and primary BC in patients < 55 years and < 65 years, respectively.

Our data are also consistent with previous literature reporting that luminal BC subtype confers an independent survival benefit regardless of tumor receptor status^25,26^.

The initial stage of disease at BC diagnosis represented an important prognostic factor for survival and is consistent with the literature on this topic^27,28^. Some preclinical and clinical studies have reported promising results for concurrent ENDO + CH in postmenopausal patients with metastatic hormone-positive BC^29,30^. In line with these findings, our patients undergoing the ENDO + CH combination showed a slight benefit in terms of mPFS with respect to those treated with ENDO + CH ± BIO. These fascinating suggestions warrant further exploration and validation prospective clinical trials.

Previous studies have shown a better prognosis for BOM patients than for those with BVM. However, in our study BOM and BVM groups had a similar mOS which may be attributable to a conditioning effect of the molecular BC subtype and also to new treatments available.

A recently published study on BOM reported no correlation between bone pain and survival. Our results are in line with these findings in both BOM and BVM groups. BM localization proved to have a prognostic value in our case series, an appendicular site negatively impacting mOS more than axial or mixed localizations. In a retrospective study conducted by Parkes et al., BOM patients with axial localisation had a mOS from the diagnosis of distant disease of 5.62 years compared to 6.78 years for those with appendicular BM and 4.58 years the appendicular + axial BM group^31^. A possible explanation for this could be the higher incidence of axial bone metastases in luminal BC.

Another interesting finding of our study was that patients with single or oligo- metastases had a better prognosis than those with multiple bone lesions. Although there is already evidence of this for the former^32^, interest in oligo-metastatic disease has recently come to the fore because of its challenging management^33,34^.

It has been seen that changes in BC cell biology occur between primary tumors and metastases^35,36^. In our study a significant difference in PgR expression was observed between primary BC and metastatic bone lesions. Clinicians should take this into account in cases of disease recurrence more than 5 years after the primary diagnosis.

Age was found to have a significant impact on OS. This is in line with retrospective works published on BC and BM patients in which age was found to be an independent prognostic factor^30^.

Finally, our data confirm the protective effect of denosumab in preventing SREs, which leveled off over time. Albeit with an incidence reducing over time.

One of the limitations of our study is the patient sample even though it’s consistent with other studies in which patients were followed prospectively in a dedicated database on BM and not extrapolated from large registries^28,32^. Another limit is the lack of information available on specific treatments, a result of opting not to enter a large amount of data into the database. To overcome this we grouped treatments into specific categories.

In contrast, the strengths of our study are its collection of prospective data on BM and their clinical evolution and the fact that it constitutes a national representative study population for this disease setting.

In conclusion, the Italian BMDB represents an invaluable tool to better understand the natural history of bone metastases from breast cancer and improve their management. The Italian BMDB continues to enroll patients also in other solid tumors to increase the case series and give more answer to clinician questions.

## Supplementary Information

Supplementary Information.

## Data Availability

The datasets gathered and analyzed during the current study are available from the corresponding author on reasonable request.
