# Supplementary material for: First prospective data on breast cancer patients from the multicentre italian bone metastasis database
Source: Sci Rep. 2021 Feb 22;11:4329. doi: 10.1038/s41598-021-83749-1 (PMC7900106; doi:10.1038/s41598-021-83749-1)
Supplement: Supplementary file 1 — Supplementary Information. [file 41598_2021_83749_MOESM1_ESM.docx]

**First prospective Data on Breast Cancer Patients from the Multicentre Italian Bone Metastasis Database**

Alberto Bongiovanni ^1^, Flavia Foca ^2^, Manuela Fantini ^3^, Maria Rosachiara Forcignanò ^4^, Fabrizio Artioli ^5^, Rossana Berardi ^6^, Enrico Campadelli ^7^, Giuseppe Procopio ^8^, Francesco Silvestris ^9^, Nada Riva ^1^, Lorena Gurrieri ^1^, Silvia Angela Debonis ^1^, Giandomenico Di Menna ^1^, Valentina Fausti ^1^, Federica Recine ^1^, Roberto Vespignani ^10^, Toni Ibrahim ^1^

^1^ Osteoncology and Rare Tumors Center (CDO-TR), IRCCS Istituto Romagnolo per lo Studio dei Tumori (IRST) "Dino Amadori", Italy

^2^ Unit of Biostatistics and Clinical Trials, IRCCS Istituto Romagnolo per lo Studio dei Tumori (IRST) "Dino Amadori", Italy

^3^ Oncology Unit, Infermi Hospital, Rimini, Italy

^4^ Oncological Unit, Vito Fazzi Hospital, Lecce, Italy

^5^ Division of Medical Oncology, Ramazzini Hospital, Carpi, Italy

^6^ Ospedali Riuniti di Ancona, Ancona, Italy

^7^ Oncology Unit, Degli Infermi Hospital, Faenza, Italy

^8^ IRCCS National Cancer Institute (INT), Milan, Italy

^9^ Department of Biomedical Science and Human Oncology, University of Bari, Bari, Italy

^10^ IT Service, IRCCS Istituto Romagnolo per lo Studio dei Tumori (IRST) "Dino Amadori", Italy

**Appendix 1**

**Data Source**

The BMDB is a database based on tailored software, established in 2014 to collect data prospectively on primary tumors and bone metastasis from patients referred by specialized cancer centers in Italy. Data entry for all healthcare providers is legally implemented, and so outcomes should reflect nationwide and international patterns of care.

All patients are enrolled consecutively at the time of the first diagnosis of bone metastasis and followed over time, until death. Because of its observational nature, all observed patients with bone metastases from solid tumors are included. The BMDB includes data common to all solid tumors and also specific information for each tumor type such as histology, mutational status, type and setting of specific treatment and stage.

Patient data are collected by independent and trained physicians, are based on individual patient clinical charts and are de-identified at the local centre as a measure to protect patient privacy.

Data are updated every 6 months by the Participating Centres (PCs) and reviewed by the Coordinator Centre (CC) to guarantee good data quality. The study complies with the ethical standards laid down in the 1964 Declaration of Helsinki and the principles of Good Clinical Practice guidelines.

To date, 15 hospitals in different Italian regions have enrolled patients in the platform. All patients are followed until death or the last visit in each center.

The BMDB platform includes the following data:

- File A: demographic information such as year of birth, place of residence and life status, death or date of last contact date if the patient was still alive.
- File B: primary tumor data: date of diagnosis, primitive tumor site, histology, grading, TNM stage [1]*,* neoadjuvant and/or adjuvant therapy and complementary radiotherapy. For Breast Cancer patients, File B also contains information on ER and PgR expression, Ki67% value, immunohistochemical c-HER2 expression or HER2 amplification by FISH according to the American Society of Clinical Oncology/College of American Pathologists (ASCO/CAPs) recommendations [2].
- File C: information on first diagnosis of bone metastasis: performance status (PS), date of appearance of bone metastasis, type of lesion, number of bone metastases, site of bone metastasis, diagnostic modality (radiological or histological), presence and date of visceral disease, type of systemic treatment. For patients with a histological diagnosis of bone metastasis, histological type and molecular information are also collected at the onset of bone metastasis. Information is also collected on type of bone targeted therapy (BTT), presence of bone pain, orthopedic surgery, radiotherapy to bone, locoregional treatment, radiometabolic treatment administered, and SREs at the onset of bone metastasis. For Breast Cancer patients, information on ER and PgR expression, Ki67% value, immunohistochemical c-HER2 expression or HER2 amplification by FISH is also registered.
- File D: data collected prospectively on disease evolution. PCs update patient outcome every six months and different data are registered on the basis of the overall response (stability, partial and complete response or progression) observed. Information on Eastern Cooperative Oncology Group Performance Status (ECOG PS) and bone-targeted therapy (BTT) are required for each type of response and in the event of disease progression, the following additional information is required: modification of type of bone lesion, number of new bone metastases, presence of bone pain, type of bone metastasis progression, site of bone metastasis, information on visceral progression, further specific systemic treatment, bone targeted therapy, orthopedic surgery, radiotherapy to bone, locoregional treatment, radiometabolic treatment, and type and date of SREs.

PS is evaluated according to the ECOG scale [3], while classification of the primary tumor site and histology are defined according to the International Classification of Diseases for Oncology, 3^rd^ Edition First Revision (ICD-O-3.1) topography codes and WHO Classification of Breast Tumors, 3^rd^ Edition [4].

Breast cancer subtypes are divided into 4 groups according to the breast cancer molecular subtype classification [5]. BM characteristics include site of metastasis, type (blastic/sclerotic, lytic, or mixed) and number of lesions at the time of diagnosis of bone metastasis. Mixed bone metastases are defined as 2 or more types of bone metastasis in the same lesion [6]. Single bone metastases are defined as one metastatic bone lesion restricted to a single site. Oligo bone metastases are defined as 2-6 lesions, including more than one lesion in the same site, while multiple bone metastases are defined as more than 6 lesions.

Sites of bone metastasis (axial or appendicular skeleton) are identified at the time of diagnosis using clinical notes and imaging reports. Bone metastases in the skull, middle ear ossicles, hyoid, rib cage, sternum, and vertebral column are classified as axial skeleton metastases, while those located in the pectoral girdle, arms, forearms, hands, pelvis, thighs, legs, feet, and ankles are classified as appendicular skeleton metastases.

There is evidence that SREs may be a clinically relevant endpoint in that they negatively patient-reported quality of life. Furthermore, SSEs, including symptomatic pathologic fractures (but not asymptomatic, radiographically identified fractures) may also be quality of life- limiting factors [6,7]. According to published data in the bone metastasis setting, SREs are classified as follows pathological fracture, hypercalcaemia, spinal cord compression, bone marrow invasion, and need for orthopedic surgery or radiotherapy.

**References**

1. Edge SB, Compton CC. The American Joint Committee on Cancer: the 7th edition of the AJCC cancer staging manual and the future of TNM. Ann Surg Oncol 2010;17(6):1471-4. https://doi.org/[10.1245/s10434-010-0985-4](https://doi.org/10.1245/s10434-010-0985-4).
2. Wolff AC, Hammond MEH, Allison KH, Harvey BE, McShane LM, Dowsett M. [HER2 Testing in Breast Cancer: American Society of Clinical Oncology/College of American Pathologists Clinical Practice Guideline Focused Update Summary](https://ascopubs.org/doi/abs/10.1200/JOP.18.00206). J Oncol Pract  2018;14(7):437-41. https//doi.org/10.1200/JOP.18.00206.
3. Oken MM, Creech RH, Tormey DC, Horton J, Davis TE, McFadden ET, et al. Toxicity and response criteria of the Eastern Cooperative Oncology Group. Am J Clin Oncol 1982;5(6):649-55.
4. Fritz A, Percy C, Jack A, Shanmugaratnam K, Sobin L, Parkin M, et al. ICD-O: International classification of diseases for oncology. 3rd ed., 1st rev. Geneva: World Health Organization; 2014.
5. Perou CM, Sorlie T, Eisen MB, van de Rijn M, Jeffrey SS, Rees CA, et al. Molecular portraits of human breast tumours. Nature 2000;406(6797):747-752.

https//doi.org/[10.1038/35021093](https://doi.org/10.1038/35021093).

1. Parkes A, Clifton K, Al‐Awadhi A, [Oke O](https://www.ncbi.nlm.nih.gov/pubmed/?term=Oke%20O%5BAuthor%5D&cauthor=true&cauthor_uid=29387785), [Warneke CL](https://www.ncbi.nlm.nih.gov/pubmed/?term=Warneke%20CL%5BAuthor%5D&cauthor=true&cauthor_uid=29387785), [Litton JK](https://www.ncbi.nlm.nih.gov/pubmed/?term=Litton%20JK%5BAuthor%5D&cauthor=true&cauthor_uid=29387785), et al. Characterization of bone only metastasis patients with respect to tumor subtypes. NPJ Breast Cancer 2018;4:2. https//doi.org/10.1038/s41523-018-0054-x.
2. [D’Oronzo S](https://www.ncbi.nlm.nih.gov/pubmed/?term=D%27Oronzo%20S%5BAuthor%5D&cauthor=true&cauthor_uid=30937279), [Coleman R](https://www.ncbi.nlm.nih.gov/pubmed/?term=Coleman%20R%5BAuthor%5D&cauthor=true&cauthor_uid=30937279), [Brown J](https://www.ncbi.nlm.nih.gov/pubmed/?term=Brown%20J%5BAuthor%5D&cauthor=true&cauthor_uid=30937279), [Silvestris F](https://www.ncbi.nlm.nih.gov/pubmed/?term=Silvestris%20F%5BAuthor%5D&cauthor=true&cauthor_uid=30937279). [Metastatic bone disease: Pathogenesis and therapeutic options: Up-date on bone metastasis management.](https://www.ncbi.nlm.nih.gov/pubmed/30937279) [J Bone Oncol](https://www.ncbi.nlm.nih.gov/pubmed/?term=Metastatic+bone+disease%3A+Pathogenesis+and+therapeutic+options%3A+Up-date+on+bone+metastasis+management) 2018;15:004-4. https//doi.org/10.1016/j.jbo.2018.10.004.

**Appendix 2**

**Role of bone biopsy**

As reported bone biopsy was performed in 58 (26.4%) cases. A significant difference in immunohistochemical characteristics between primary tumor and BM was found with regard to PgR expression. Of the 33 patients PgR-positive at diagnosis, 45.5% showed PgR-negativity at BM biopsy (p= 0.002) (data not shown)

**Solitary bone metastases**

Solitary bone lesions were present in 34 (16.0%) patients, 26 (76.4%) of whom had an axial metastasis to the spine and 8 (23.5%) a metastasis to the appendicular skeleton. Ten (37.0%) patients reported pain at the time of the diagnosis of the metastasis, while 17 (63.0%) were asymptomatic for pain. The pain status of 7 patients was unknown. Ten (37.0%) patients had at least one documented SRE at diagnosis. Twenty-two patients had undergone pharmacological treatment for bone disease (19 with zoledronic acid and 3 with denosumab). Median duration of treatment with zoledronic acid was 31 months (interquartile range: 13-49). Twenty-two (64.7%) patients had undergone endocrine therapy, alone or in association with chemotherapy or biotherapy. Time to progression for the bone-only group was 45.9 months (65%CI: 7.5-not estimable), while time to visceral or bone progression was 12.8 months (95%CI: 7.5-18.3). The overall survival of this subgroup was 257 months (95%CI:143.0-not estimable).

Solitary lesions were more frequently associated with metachronous than synchronous BM (85.3% *vs*. 14.7%) (p = 0.013).

**Bone metastases characteristics**

Bone-targeted therapy was administered to 156 (70.9%) patients, more often in those with BOM (54.2%) than with BVM (45.8%) (p = 0.052).

Axial localization was more common in patients with metachronous BM (85.5%, *vs*. 14.5%). Vertebral and appendicular localizations were more frequent (53.1%) in cases of metachronous BM with respect to primary tumor (p = 0.003), while patients with both axial and appendicular localizations showed a similar distribution between synchronous and metachronous BM. BM were prevalently lytic (60.2%) (Tables 3 and 4 supplementary data).

**SREs and bone metastases from breast cancer**

Forty-six (32.2%) pathological fractures, 86 (60.1%) radiotherapy, 7 (4.9%) surgery and 4 (2.8%) spinal cord compression occurred during the study. SRE incidence one year after diagnosis was 13.6% (*n* = 30), with a decreased percentage in the following years: 4.5% (*n* = 10), 5.0% (*n* = 11) and 3.1% (*n* = 7) at 2, 3 and 4 years after the first diagnosis of bone metastasis. Of 98 patients with at least one SRE, 40 (18.1%) had only one SRE at diagnosis, 33 (15.0%) had one or more SREs 3 three months after diagnosis and 25 (11.4%) had a SRE both at diagnosis and during the follow-up period. The cumulative incidence rate of the first SRE during follow-up was 14.2% (95%CI:10.1-19.7) and was similar when the following variables were considered: synchronous or metachronous BM, presence of bone-only metastasis *vs*. bone metastasis+visceral metastasis, type of first-line treatment, number of bone lesions, type of bone metastasis and presence of pain at diagnosis (data not shown).

**Supplementary figure 1: Bone Disease free interval by age**


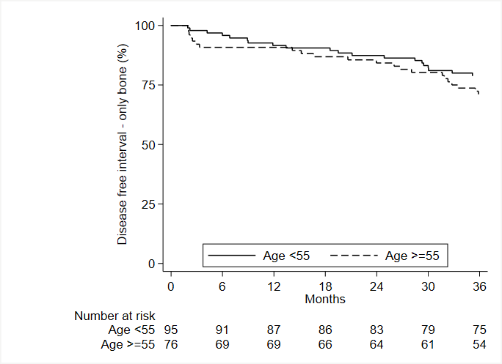


**Supplementary figure 2: Overall survival from metastatic diagnosis by stage (a) and by histological subtype (b)**


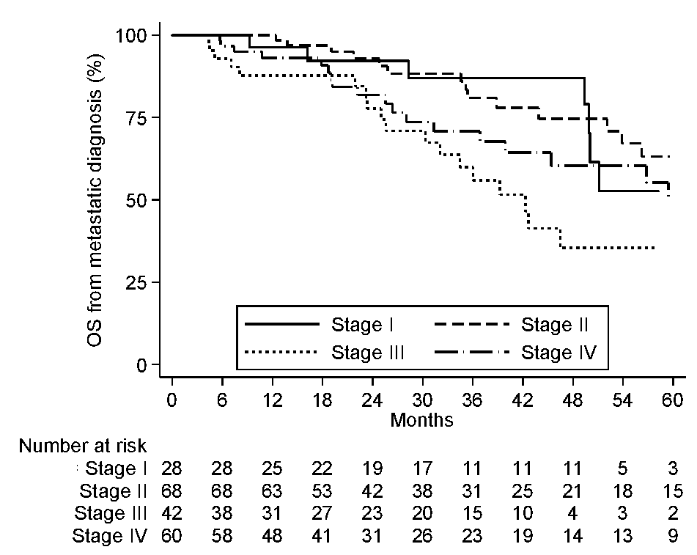

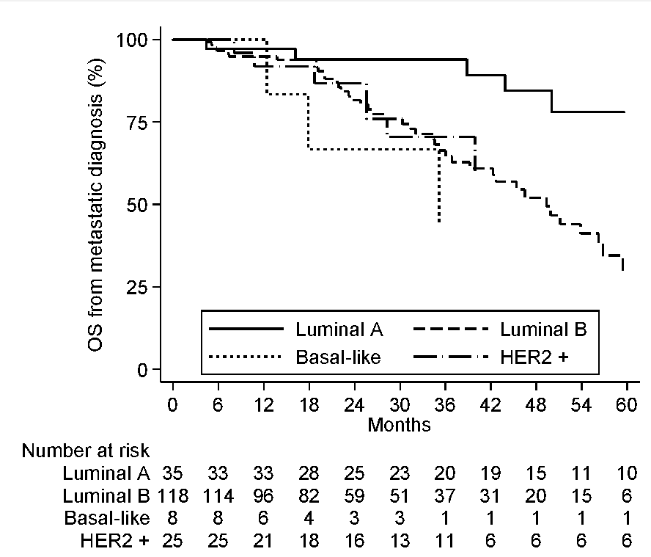


**Supplementary figure 3: Progression free survival by therapy**


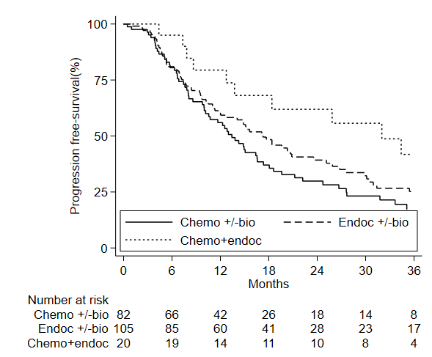


**Supplementary figure 4: Cumulative incidence by bone target therapy type**


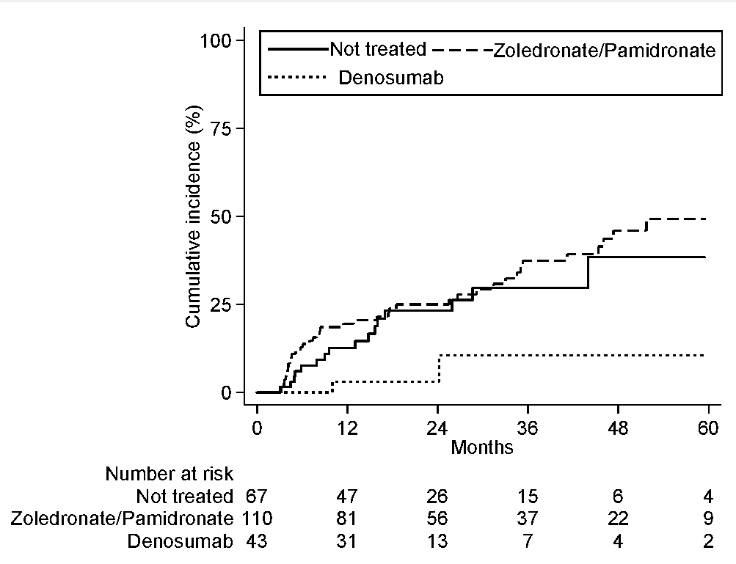


**Table 1 (supplementary data)**

List of participating centers

| **CENTER** | **Date of study approval** | **Code** |
| --- | --- | --- |
| IRST – Romagna Sub-region | 20/03/2014 | Prot. 1783/2014 |
| Roma Unicampus | 22/07/2014 | Prot. 24/14 OSS |
| Lecce | 09/10/2014 | Verb. N.16 |
| Bari Policlinico Universitario | 17/12/2017 | Prot. 46/CE |
| Carpi | 03/02/2015 | Prot. 410/CE |
| Napoli Pascale | 21/01/2015 | Not available |
| Taormina | 26/01/2015 | Verb. 2/2015 |
| Imola | 23/02/2015 | Cod. CE 14132 |
| Negrar | 08/04/2015 | 493 CESC |
| Piacenza | 22/05/2015 | Rep. N. 1467/2015 |
| Cuneo | 17/12/2014 | Prot. 38247 |
| Napoli Cardarelli | 28/05/2015 | Verb. 7/15 |
| Reggio Emilia | 20/01/2015 | Prot. 2015/2969 |
| Treviglio | 23/04/2015 | Not available |
| Benevento | 18/05/2015 | CECN/214 |
| Pavia | 17/11/2014 | Prot. N. 20140036231 |
| Belcolle | 01/04/2015 | Prot. 655 |
| Genova | 12/02/2015 | Reg. n. 447REG2014 |
| Ferrara | 26/03/2015 | Not available |
| Roma reg. Elena | 13/10/2015 | CEC/951/15 |
| Milano INT | 22/09/2015 | Not available |
| Ancona | 26/02/2016 | Prot.2016-0045 OR |
| Legnano | 02/10/2015 | Reg. 494-102015 |
| Bari IRCCS | 27/10/2015 | 550/CE |
| Grottaferrata | 04/12/2018 | Prot. 55592 |
| Aviano | 20/11/2018 | CEUR-2018-OS-127-CRO |
| padova IOV | 15/07/2019 | 2019/71 |
| Brescia | 11/12/2018 | Not available |

**Table 2 (supplementary data)**

Median OS and independent risk factors for OS.

|  | **OS** | | | **OS from diagnosis of metastatic disease** | | |
| --- | --- | --- | --- | --- | --- | --- |
|  | **Median**  **(95%CI)** | **HR from univariate Cox regression model (95%CI)** | **HR from multivariate Cox regression model (95%CI)** | **Median**  **(95%CI)** | **HR from univariate Cox regression model (95%CI)** | **HR from multivariate Cox regression model (95%CI)** |
| **All cases** | 217.5 (172.5-340.1) | - | - | 66.8 (52.1-79.2) | - | - |
| **Age at diagnosis of primary BC, years** |  |  |  |  |  |  |
| <55 years | 340.1 (229.5-NE) | 1.00 | 1.00 | - | - | - |
| ≥55 years | 128.1 (101.2-182.6) | 2.85 (1.7-4.7) | 2.92 (1.4-6.0) | - | - | - |
| **Age at diagnosis of BM, years** |  |  |  |  |  |  |
| <65 | - | - | - | 66.8 (50.0-NE) | 1.00 | - |
| ≥65 | - | - | - | 63.1 (49.4-79.2) | 1.12 (0.7-1.7) | - |
| **BC molecular subtypes** |  |  |  |  |  |  |
| Luminal A | NE | 1.00 | 1.00 | NE | 1.00 | 1.00 |
| Luminal B | 128.1 (108.0-182.6) | 3.76 (1.5-8.9) | 4.10 (1.5-11.1) | 49.4 (39.2-56.8) | 3.96 (1.6-9.3) | 3.67 (1.5-8.7) |
| Basal-like | 101.2 (17.1-NE) | 5.2 (1.2-21.3) | 0.47 (0.1-4.7) | 35.1 (12.4-NE) | 3.29 (0.8-13.4) | 3.20 (0.7-13.5) |
| HER2+ | 274.5 (70.3-NE) | 2.74 (0.9-7.9) | 1.73 (0.5-5.8) | 70.0-28.2-NE) | 2.57 (0.9-7.2) | 2.92 (1.0-8.2) |
| **Stage at diagnosis** |  |  |  |  |  |  |
| I | 274.5 (202.1-NE) | 1.00 | 1.00 | 106.0 (49.9-NE) | 1.00 | 1.00 |
| II | 340.1 (55.9-208.6) | 0.94 (0.4-2.1) | 1.28 (0.4-4.0) | 81.9 (53.8-NE) | 0.84 (0.36-1.95) | 0.99 (0.4-2.4) |
| III | 117.8 (98.0-NE) | 2.9 (1.2-6.9) | 2.58 (0.7-8.3) | 42.3 (30.3-NE) | 2.54 (1.1-5.9) | 2.32 (0.9-5.7) |
| IV | 65.3 (41.0-80.9) | 9.84 (4.0-24.0) | 8.69 (2.6-28.9) | 59.4 (39.9-72.9) | 1.55 (0.6-3.5) | 1.45 (0.6-3.4) |
| **BM** |  |  |  |  |  |  |
| Lytic | 182.5 (128.1-252.5) | 1.00 | - | 63.1 (49.9-91.9) | 1.00 | - |
| Osteoblastic | 274.5 (229.5-NE) | 0.64 (0.3-1.2) | - | 56.3 (34.5-NE) | 1.21 (0.65-2.2) | - |
| Mixed | NE | 0.64 (0.2-1.3) | - | 72.9 (56.8-NE) | 0.60 (0.2-1.2) | - |
| **Site of first BM** |  |  |  |  |  |  |
| Axial +/- other | 252.5 (182.5-343.0) | 1.00 | 1.00 | 66.8 (52.1-106.0) | 1.00 | - |
| Appendicular +/- other | 157.6 (41.0-NE) | 1.54 (0.6-3.4) | 2.35 (0.8-6.6) | 79.1 (19.1-NE) | 1.53 (0.6-3.4) | - |
| Both + other | 217.5 (100.9-NE) | 1.65 (1.0-2.6) | 2.20 (1.1-4.6) | 59.4 (43.8-NE) | 1.24 (0.7-2.0) | - |
| **Pain at diagnosis** |  |  |  |  |  |  |
| Yes | 143.8 (98.0-274.5) | 1.00 | 1.00 | 56.8 (42.3-72.9) | 1.00 | - |
| No | 257.4 (135.1-NE) | 0.56 (0.3-0.9) | 0.49 (0.2-0.9) | 66.8 (45.4-NE) | 0.67 (0.4-1.1) | - |
| **Systemic treatment after first diagnosis of BM** |  |  |  |  |  |  |
| CH+/-BIO | 135.1 (102.9-257.4) | 1.00 | 1.00 | 59.4 (42.7-72.9) | 1.00 | - |
| ENDO+/-BIO | 252.5 (202.1-NE) | 0.52 (0.3-0.8) | 0.40 (0.2-0.8) | 72.4 (52.1-106.0) | 0.72 (0.4-1.1) | - |
| CH+ENDO | NE | 0.92 (0.3-2.2) | 0.39 (0.1-1.2) | 56.8 (36.0-NE) | 0.91 (0.3-2.1) | - |

OS, overall survival; HR, hazard ratio; BC, breast cancer; NE, not evaluable from statistical software; BM, bone metastases; CH, chemotherapy; BIO, biological therapy; ENDO, endocrine therapy

**Table 3 (supplementary data)**

Median time to disease and bone progression and independent risk factors of progression

|  | **PFS** | | **bPFS** | |
| --- | --- | --- | --- | --- |
|  | **Median (95%CI)** | **HR from univariate Cox regression model (95%CI)** | **Median (95%CI)** | **HR from univariate Cox regression model (95%CI)** |
| **All cases** | 15.1 (12.6-18.4) | - | 45.9 (30.8-63.0) | - |
| **Age at diagnosis of BM, years** |  |  |  |  |
| <65 | 14.9 (11.7-18.3) | 1.00 | 66.6 (34.4-107.4) | 1.00 |
| ≥65 | 15.6 (11.3-20.9) | 1.05 (0.7-1.4) | 34.5 (19.6-46.4) | 1.51 (1.1-2.1) |
| **Metastasis** |  |  |  |  |
| Synchronous bone | 15.1 (11.1-22.2) | 0.98 (0.6-1.4) | 45.9 (18.9-NE) | 0.90 (0.5-1.4) |
| Metachronous bone | 15.0 (12.2-18.3) | 1.00 | 42.3 (29.8-66.7) | 1.00 |
| **Type of metastasis** |  |  |  |  |
| BOM | 18.0 (14.3-25.1) | 0.81 (0.5-1.1) | 45.9 (30.3-59.4) | 1.07 (0.7-1.5) |
| VBM | 12.7 (9.7-16.6) | 1.00 | 51.7 (21.8-70.1) | 1.00 |
| **BM** |  |  |  |  |
| Lytic | 16.5 (12.2-26.7) | 1.00 | 52.1 (30.9-66.8) | 1.00 |
| Osteoblastic | 13.4 (7.7-22.2) | 1.30 (0.8-1.9) | 29.8 (16.3-NE) | 1.19 (0.7-1.9) |
| Mixed | 17.3 (11.7-25.1) | 1.0 (0.6-1.6) | 56.8 (32.1-NE) | 0.80 (0.4-1.4) |
| **No. of BM** |  |  |  |  |
| 1-6 | 14.0 (9.7-18.0) | 1.00 | 66.7 (35.1-81.9) | 1.00 |
| >6 | 16.6 (13.8-23.6) | 1.04 (0.8-1.2) | 34.4 (25.0-51.7) | 1.04 (0.8-1.2) |
| **Pain at diagnosis** |  |  |  |  |
| Yes | 15.0 (11.3-20.9) | 1.02 (0.7-1.4) | 39.9 (25.0-56.8) | 0.90 (0.5-1.3) |
| No | 16.5 (11.1-18.3) | 1.00 | 35.9 (21.2-81.9) | 1.00 |
| **Systemic treatment after first diagnosis of BM** |  |  |  |  |
| CH+/-BIO | 13.4 (10.0-16.6) | 1.00 | 51.7 (30.3-72.8) | 1.00 |
| ENDO+/-BIO | 17.3 (12.0-23.6) | 0.81 (0.5-1.1) | 38.2 (21.9-66.7) | 1.25 (0.8-1.8) |
| CH+ENDO | 32.0 (12.7-NE) | 0.51 (0.2-0.9) | 56.8 (23.3-NE) | 1.01 (0.5-2.0) |
| **SRE at diagnosis** |  |  |  |  |
| Yes | 15.1 (11.0-26.7) | 0.84 (0.6-1.1) | 59.4 (42.3-81.9) | 1.00 |
| No | 14.9 (11.6-18.3) | 1.00 | 34.6 (23.4-52.1) | 0.73 (0.4-1.10) |

PFS, progression-free survival; bPFS, bone progression-free survival; BOM, bone-only metastasis; VBM, visceral and bone metastasis; CH, chemotherapy; BIO, biological therapy; ENDO, endocrine therapy; SRE, skeletal-related event

**Table 4 (supplementary data)**

Bone metastasis characteristics in relation to presence of non skeletal metastases.

| **Characteristics of first BM** | **No. patients**  **(n = 218)** | **BOM (n = 109)**  **No. (%)** | **BVM (n = 109)**  **No. (%)** | **p-value** |
| --- | --- | --- | --- | --- |
| **Site of first BM** |  |  |  |  |
| Axial +/- other | 116 (54.5) | 56 (48.3) | 60 (51.7) |  |
| Appendicular +/- other | 19 (8.9) | 8 (42.1) | 11 (57.9) | 0.586 |
| Both + other | 78 (36.6) | 42 (53.9) | 36 (46.1) |  |
| **No. BM** |  |  |  |  |
| 1 | 33 (15.7) | 17 (51.5) | 16 (48.5) | 0.779 |
| 2-6 | 58 (27.6) | 27 (46.5) | 31 (53.5) |  |
| >6 | 119 (56.7) | 62 (52.1) | 57 (47.9) |  |
| **Type of bone lesion** |  |  |  |  |
| Lytic | 111 (60.3) | 53 (47.7) | 58 (52.3) | 0.383 |
| Osteoblastic | 44 (23.9) | 23 (52.3) | 21 (47.7) |  |
| Mixed | 29 (15.8) | 18 (62.1) | 11 (37.9) |  |
| Unknown | 34 | 15 | 19 |  |
| **Pain at diagnosis** |  |  |  |  |
| Yes | 96 (56.1) | 55 (57.3) | 41 (42.7) | 0.120 |
| No | 75 (43.9) | 34 (45.3) | 41 (54.7) |  |
| Unknown | 47 | 20 | 27 |  |
| **SRE at diagnosis** |  |  |  |  |
| Radiotherapy | 47 (68.1) | 25 (53.2) | 22 (46.8) |  |
| Pathological fracture | 17 (24.6) | 10 (58.8) | 7 (41.2) | - |
| Surgery | 4 (5.8) | 4 (100.0) | 0 (0.0) |  |
| Spinal cord compression | 1 (1.5) | 1 (100.0) | 0 (0.0) |  |
| **Type of SRE at diagnosis** |  |  |  |  |
| Radiotherapy | 47 (68.1) | 25 (53.2) | 22 (46.8) | 0.240 |
| Other | 22 (31.9) | 15 (68.2) | 7 (31.8) |  |
| **Treatment after diagnosis of first BM** |  |  |  |  |
| CH +/-BIO | 80 (38.7) | 29 (36.2) | 51 (63.8) | 0.001 |
| ENDO +/-BIO | 105 (50.7) | 66 (62.9) | 39 (37.1) |  |
| CH+ENDO | 20 (9.7) | 8 (40.0) | 12 (60.0) |  |
| Other | 2 (0.9) | 0 (0.0) | 2 (100.0) |  |
| No | 11 | 6 | 5 |  |
| **Bone-targeted therapy** |  |  |  |  |
| No | 64 (27.5) | 25 (39.7) | 38 (60.3) | 0.052 |
| Yes | 156 (71.1) | 84 (54.2) | 71 (45.8) |  |
| Zoledronic acid | 105 (69.1) | 58 (55.2) | 47 (44.8) |  |
| Denosumab | 43 (28.3) | 23 (53.5) | 20 (46.5) |  |
| Pamidronate | 4 (2.6) | 3 (75.0) | 1 (25.0) |  |
|  |  |  |  |  |

BM, bone metastasis; BOM, bone-only metastasis; BVM, visceral and bone metastasis; SRE, skeletal-related event; CH, chemotherapy; BIO, biological therapy; ENDO, endocrine therapy

**Table 5 (supplementary data)**

BM characteristics in relation to presence of synchronous or metachronous metastases.

| **Characteristics of first BM** | **No. patients**  **(n = 220)** | **Synchronous BM**  **(n = 49)**  **No. (%)** | **Metacronous BM**  **(n = 171)**  **No. (%)** | **p-value** |
| --- | --- | --- | --- | --- |
| **Site of first BM** |  |  |  |  |
| Axial +/- other | 117 (54.4) | 17 (14.5) | 100 (85.5) |  |
| Appendicular +/- other | 19 (8.8) | 4 (21.1) | 15 (78.9) | 0.003 |
| Both + other | 79 (36.8) | 28 (35.4) | 51 (64.6) |  |
| **No. of BM** |  |  |  |  |
| 1 | 34 (16.0) | 5 (14.7) | 29 (85.3) | 0.013 |
| 2-6 | 58 (27.4) | 7 (12.1) | 51 (87.9) |  |
| >6 | 120 (56.6) | 36 (30.0) | 84 (70.0) |  |
| **Type of bone lesion** |  |  |  |  |
| Lytic | 112 (60.2) | 28 (25.0) | 84 (75.0) | 0.618 |
| Osteoblastic | 45 (24.2) | 8 (17.8) | 37 (82.2) |  |
| Mixed | 29 (15.6) | 7 (24.1) | 22 (75.9) |  |
| Unknown | 34 | 6 | 28 |  |
| **Pain at diagnosis** |  |  |  |  |
| Yes | 97 (56.4) | 26 (26.8) | 71 (73.2) | 0.210 |
| No | 75 (43.6) | 14 (18.7) | 61 (81.3) |  |
| Unknown | 48 | 9 | 39 |  |
| **SRE at diagnosis** |  |  |  |  |
| Radiotherapy | 47 (68.1) | 9 (19.1) | 38 (80.9) |  |
| Pathological fracture | 17 (24.6) | 5 (29.4) | 12 (70.6) | - |
| Surgery | 4 (5.8) | 0 (0.0) | 4 (100.0) |  |
| Spinal cord compression | 1 (1.5) | 0 (0.0) | 1 (100.0) |  |
| **Type of SRE at diagnosis** |  |  |  |  |
| Radiotherapy | 47 (68.1) | 9 (19.1) | 38 (80.9) | 0.755 |
| Other | 22 (31.9) | 5 (22.7) | 17 (77.3) |  |
| **Treatment after diagnosis of first BM** |  |  |  |  |
| CH +/-BIO | 82 (39.3) | 23 (28.1) | 59 (71.9) | 0.124 |
| ENDO+/-BIO | 105 (50.2) | 19 (18.1) | 86 (81.9) |  |
| CH+ENDO | 20 (9.6) | 7 (35.0) | 13 (65.0) |  |
| Other | 2 (0.9) | 0 (0.0) | 2 (100.0) |  |
| No | 11 | 0 | 11 |  |
| **Bone target therapy** |  |  |  |  |
| No | 64 (29.1) | 16 (25.0) | 48 (75.0) | 0.533 |
| Yes | 156 (70.9) | 33 (21.1) | 123 (78.9) |  |
| Zoledronic acid | 106 (69.3) | 23 (21.7) | 83 (78.3) |  |
| Denosumab | 43 (28.1) | 10 (23.3) | 33 (76.7) |  |
| Pamidronate | 4 (2.6) | 0 (0.0) | 4 (100.0) |  |

BM, bone metastasis; SRE, skeletal-related event; CH, chemotherapy; BIO, biological therapy; ENDO, endocrine therapy

**Supplementary figure 1 Disease-free interval by age in primary tumour.**

**Supplementary figure 2. Overall survival from metastatic disease by (A) molecular subtype and (B) stage at primary diagnosis.**

**Supplementary figure 3. Progression free survival by treatment.**

**Supplementary figure 4. Cumulative incidence of SREs by pharmacological treatment for bone metastases.**
